# Nonlinear mechanical response analysis and convolutional neural network enabled diagnosis of single-span rotor bearing system

**DOI:** 10.1038/s41598-024-61180-6

**Published:** 2024-05-06

**Authors:** Bing Qian, Yinhui Cai, Yinkang Ran, Weipeng Sun

**Affiliations:** 1 CHN Energy Dadu River Repair & Installation Co., Ltd., Leshan, 614900 China; 2 Institute of Water Resources and Hydroelectric Engineering, Xi’an University of Technology, Xi’an, 710048 China

**Keywords:** Single-span rotor bearing system, Spindle bending, Spindle crack, Convolutional neural network machine learning, Energy science and technology, Engineering

## Abstract

The wide application of rotating machinery has boosted the development of electricity and aviation, however, long-term operation can lead to a variety of faults. The use of different measures to deal with corresponding malfunctions is the key to generating benefits, so it is significant to carry out the fault diagnosis of rotating machinery. In this work, a test bench for single-span rotor bearings was established, three faults, including spindle bending, spindle crack without end loading and spindle crack with end loading, are experimental analyzed with basic mechanical response. Moreover, a diagnosis is performed using a convolutional neural network, according to the differences in mechanical responses of the three faults obtained from experiments. For three faults, the change in the properties of spindle itself results in different axis trajectories and spectra. Compared with spindle bending fault, spindle crack fault not only cause 1×, 2×, 3× frequency component excitation, also 4×, 5× frequency component excitation. Additionally, the classification accuracy of the training set and the test set under machine learning for the three types of working conditions is 100%. This indicates that the network can significantly identify signal features so as to make effective fault classification.

## Introduction

Rotating machinery, as an indispensable facility in the industry, is applied in various fields such as petrochemical, power, metallurgy, aerospace and so on^1–3^. The vibration fault occurrence of rotating machinery equipment has extremely bad consequences^4,5^. With the upgrading of the national industrial system and the increasing production requirements, the requirements for rotating machinery are also constantly increasing. However, with the increasing demand for equipment industry, vibration faults in rotating machinery have become a major obstacle to the industrial development process^6–8^.

Due to the harsh working environment or changes in load, as well as various other reasons, rotating machinery equipment may experience faults, such as rotor imbalance, spindle misalignment, and loose foundation^9,10^. These faults cause radial vibration, axial vibration, and torque vibration in the system, among which radial vibration is the most likely to occur. The vibration mechanical responses are an important reference for mechanical fault diagnosis^11,12^. Guo et al.^13^. successfully identified the misalignment and friction faults of the rotor through the purified axis trajectory. Miao et al.^14^. combined binocular stereo vision to reconstruct the three-dimensional axis trajectory. Based on the linear relationship between phase and displacement, the actual axis trajectory can be obtained for more accurate fault diagnosis. Liu et al.^15^ established a dynamic model of unsteady oil film force for the rotor stator bearing system. And by numerical methods, they pointed out that the change of oil film velocity is closely related to oil film force. Wang et al.^16^ proposed a uniform two-phase flow model to calculate the axial trajectory of radial bearings in twin screw compressors. The influence of refrigerant mass fraction and structural parameters at the supply tank was theoretically analysed. Zhou et al.^17^ explored the dynamic characteristics of rotor sealed bearing system by nonlinear methods such as bifurcation diagrams. Lin et al.^18^investigated the dynamic characteristics of the system and inverse eccentricity when sliding bearing bracket is loosened using nonlinear analysis. They found that the loosening fault showed a multi-frequency or continuous spectrum, and the rotor trajectory is “cylindrical”.

Traditional vibration signal recognition is achieved through human–machine dialogue, and which are not sufficiently accurate and intelligent^19–21^. However, machine learning has greatly improved the speed, accuracy and confidence in recognizing fault diagnosis in rotating systems^22–24^. Shubita et al.^25^ proposed a workflow for constructing a fault diagnosis system based on acoustic emission (AE) using machine learning (ML) technology. Which is applied for real-time fault detection and classification in rotating machines, and this method achieved an accuracy of 96.1%. Lee et al.^26^ used Support Vector Machines (SVM) to identify defects, but the defects can be quickly identified only from vibration data of normal and abnormal states. Zhang et al.^27^ proposed a new fault diagnosis method that combines SVMD entropy with machine learning. The combination of SVMD entropy and machine learning is more effective in fault diagnosis of rotating machinery through more effective fault feature vector selection. Kumar et.al.^28^ used experiments to obtain faulty datasets and further compared the classification accuracy of machine learning models such as ANN and CNN. Jablon et al.^29^ proposed a new strategy that utilizes machine learning strategies based on vibration trajectory features to improve the diagnostic performance of rotating machinery. this method had an accuracy of nearly 60%. Manikandan et al.^30^ investigated the advantages and advanced modes of deep neural networks applied to multi component fault diagnosis. Finally, different algorithms were proposed to improve the quality of fault diagnosis, and research ideas for applying machine learning methods to various rotating equipment were summarized. Inyang et al.^31^ proposed a comprehensive learning method with optimizing signal processing transforms for single and multiple fault diagnosis of different rotating machinery components. For the multiple fault diagnosis of these components, aspects such as optimized bicoherence and deep hybrid ensemble learning are explored. Moreover, Ma et al.^32^ used a multi-objective optimization algorithm (including Convolutional Residual Network (CRN), Deep Belief Network (DBN), and Deep Auto-Encoder (DAE)) as an integration strategy and further proposed an integrated deep learning diagnosis method.

Reviewing the aforementioned studies, most research on rotor-bearing systems has focused on failures arising during operation, such as misalignment, unbalance, and oil-film instability. However, the vibration response of rotating system caused by insufficient manufacturing of the shaft itself and failures occurring during long-term operation, such as bends and cracks, is not well defined, and there is also a lack of rationale and methodology for categorizing such failures. On the other hand, the characteristics of rotor-bearing systems caused by various types of faults are ultimately reflected in vibration signals, which may be reflected in single or multiple parameters such as amplitude, dominant frequency, and high-octave frequency, and the accuracy of using machine learning methods such as BP and SVM to determine the similar characteristics of these different faults is insufficient. Therefore, it is significant to employ Convolutional Neural Network (CNN) to classify higher-level features and capture higher-level semantic information present in rotating systems. In this paper, three faults of spindle bending, spindle cracks without end loading and spindle cracks with end loading, are experimentally tested and analysed with basic vibration mechanical response and CNN machine learning diagnosis. The research point of this paper focuses on vibration difference analysis and machine learning recognition under three types of faults that all generate spindle bending. Based on the similar vibration responses induced under three faults, they are accurately diagnosed and classified by using CNN.

## Single-span rotor bearing system and experiment setting

**Figure 1:**
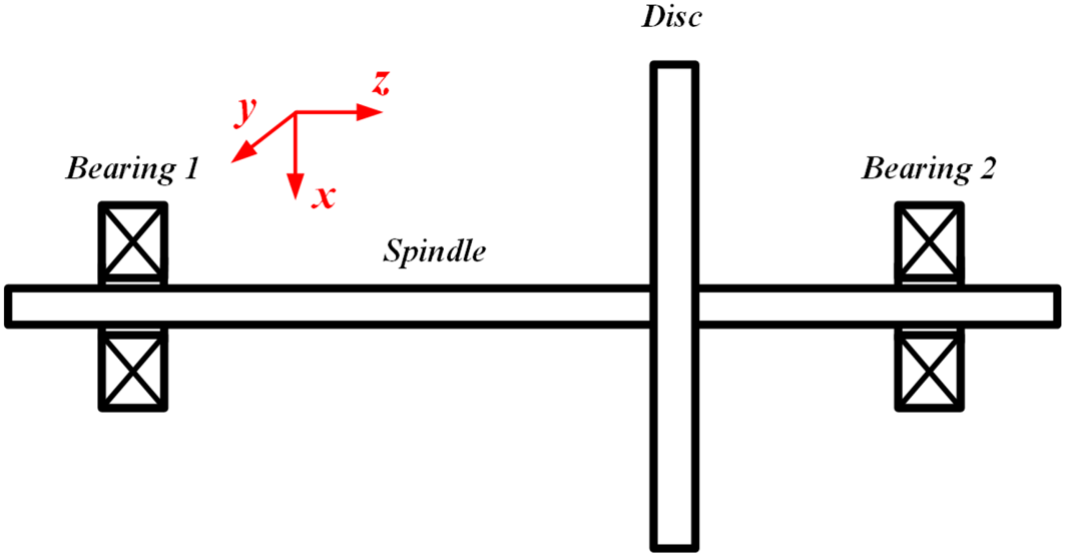
The schematic diagrams of single-span rotor bearing system.

**Figure 2:**
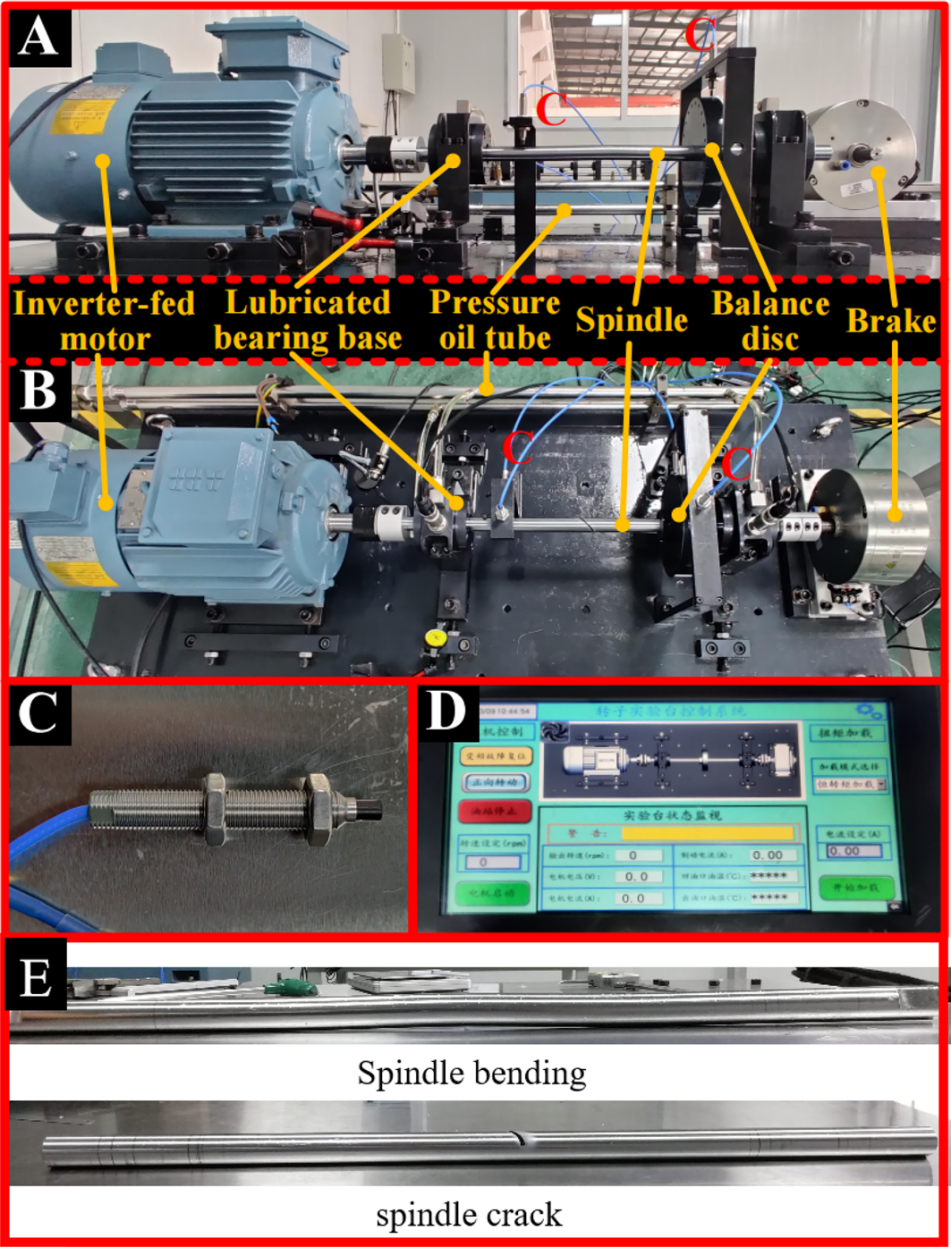
The schematic of the diagnosis bearing testing platforms for (**a**) spindle bending and (**b**) spindle crack. (**c**) The schematic of eddy current displacement sensor and (**d**) experiment platform’s intelligent electrical control system, (e) physical drawing of spindle bending and spindle cracking.

The rotating machinery can be simplified as a single-span rotor bearing system, which generally consists of rotor disc, spindle and bearings, as shown in Fig. 1. The two ends of the shaft can be externally connected with force applying machinery to provide a continuous source of energy for the system, and the main working components are installed on the shaft. In order to obtain characteristic data of spindle bending and crack diagnosis, experimental platforms, as shown in Fig. 2, are built to extract the values of spindle displacement in the horizontal and vertical directions. From Fig. 2a and b, the experimental platform contains six components, including inverter-fed motor, lubricated bearing bases, pressure oil tube, spindle, balance disc and brake. Among them, the spindle, as the main research object, is supported by the left and right lubricated bearing bases at a certain height. A balance disc is installed on the right side of the spindle to simulate the essential working components in the shaft system, such as the rotor of a steam turbine, to improve the authenticity and reliability of the data. The enough space is leaved in the middle of the shaft to apply fault features, like the bending as shown in Fig. 2a and crack as shown in Fig. 2b. The lubricated bearing base contains one lubricating bearing inside. And a certain amount of lubricating oil is delivered from the rear pressure oil tube into them to ensure effective rotation of the spindle during operation. Besides, the lubricated bearing bases are all installed on a cast iron basic platform, which weighs 373.825 kg to ensure the elimination of vibration caused by mechanical equipment other than the bearing system during the experiment. An inverter-fed motor with a power of 3 kW is equipped at the left side of the system to drive the shaft. It can reach a rotating speed of 2500 rpm. Meanwhile, the brake equipped is used for safe braking and maintaining stable experimental speed. By adjusting the applied current, the brake can apply a certain radial load to the shaft. The inverter-fed motor and brake are connected to the spindle through two diaphragm couplings.

As shown in Fig. 2e, spindle bending usually refer to thermal deformation of rotor shaft, which is mostly generated by asymmetric temperature difference of the rotor shaft cross-section during system operation, and spindle bending is one of the common failures in rotor-bearing systems. Bending was performed at shaft center position. In order to achieve spindle bending diagnosis, the experimental spindle is machined with processed with a bending part with a length of 19 mm and a deviation of 2 mm from the axis centre. Bending can be seen as evenly distributed along the middle of the axis to both sides, for spindle bending shafts, the maximum bending degree corresponds to the key-phase position when it is positively upward. For crack diagnosis, a groove with a width of 2.06 mm, a depth of 10 mm, and an angle of 45° with the axis centre is machined in the middle of the experimental spindle, crack angle is 90° cis-turned from key-phase position. The value of crack depth is equal to the axis radius. The displacement data are collected through eddy current sensors (RSW-3300), as shown in Fig. 2c. They are installed at the near the left lubricated bearing base and the outer side of the balance disc in the horizontal and vertical directions. The experiment platform is controlled and monitored by the HD-FD-H-03 intelligent electrical operation system as shown in Fig. 2d. The intelligent electrical operation system is able to achieve the operations of the rotation speed change of the rotor bearing system, forward and reverse rotation of spindle and so on. The size and physical parameters of the main components of the experiment testing system are given in the Table 1. During testing experiment, the outside condition is close to environmental temperature and pressure. However, the oil pressure inside the lubricated bearing bases maintains at 0.2 MPa. The testing rotating speed increases from 300 to 2500 rpm with the interval of 100 rpm.

**Table 1:** Experimental parameters of the single-span rotor bearing system.

Parameters	Value
The length of spindle	550 mm
The mass of the steel balance disc	2.9 kg
The diameter of spindle	20 mm
Young’s modulus of spindle	210 × 10^9^ Pa
The inner diameter of cylindrical lubricated bearing	10.00 mm
End loading	0.359 N/m

## Result

Real-time monitoring of faults refers to controlling the process of faults from the absence to presence. But in this work, fault results are the object of monitoring, and the goal of study is also the various vibration characteristics of the faults under different operating conditions, so it is necessary to make the system show the corresponding faults under specific conditions.

In this paper, the vibration characteristics of three types of faults, spindle bending, spindle cracks without end loading and spindle cracks with end loading, that frequently occur in the rotor bearing system were analysed through axis trajectory and spectrum diagram. For spindle crack faults, we also studied the effect of axial end load on vibration of rotor bearing system. Finally, the different faults are classified and analysed by machine learning method of convolutional neural network.

### Mechanical response of spindle bending

**Figure 3:**
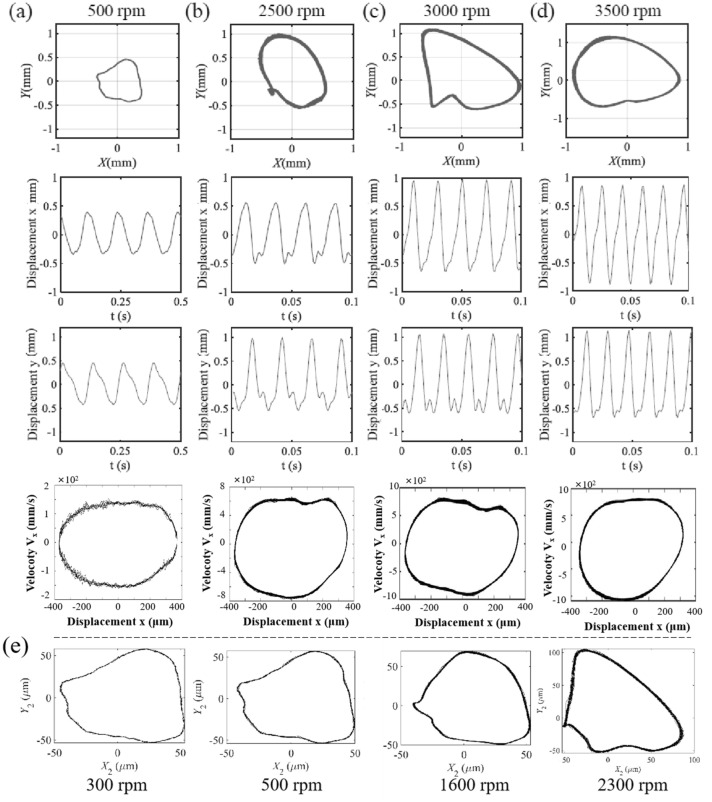
The axis trajectory, time history curve in *x*, *y* direction and vibration velocity in *x* direction of single-span rotor bearing system with spindle bending under (**a**) 500 rpm, (**b**) 2500 rpm, (**c**) 3000 rpm and (**d**) 3500 rpm, (**e**) axial trajectories at lower rotor speeds (300 rpm, 500 rpm, 1000 rpm, 1600 rpm, 2300 rpm).

Figure 3 depicts the axis trajectory and time history curve in *x*, *y* direction of single-span rotor bearing system with spindle bending under 500 rpm, 2500 rpm, 3000 rpm and 3500 rpm. It can be seen that the axis trajectory exhibits non circular or elliptical trajectory so as to show the obvious system instability under spindle bending fault. When the rotor speed is relatively low (300–2300 rpm), the axis trajectory presents an approximate quadrilateral shape, which is shown in Fig. 3e. The significant changes of operating state, velocity and acceleration, of the axis mainly occur at the corners of the quadrilateral trajectory. Thus, the time history curves of the axis in the *x* and *y* directions are closer to the serrated shape. The disturbances at the corners of the quadrilateral trajectory caused by rotor bearing imbalance exhibits relatively obvious superimposed wave phenomenon on the peak and valley positions of time history curve. As the rotating speed increases, the maximal displacement of the axial increases, but the trajectory gradually smooths out. Although the main body of the axis trajectory appears elliptical, the spline bending results in the appearance of a local 8-shaped trajectory when the rotating speed is about 2500 rpm as shown in Fig. 3b. The maximal displacement of the axial at *y* direction obviously increases when rotating speed increases between 300 and 2500 rpm. And it increases to its maximum value with rotating speed of 2500 rpm.

As the rotating speed further increases, local 8-shaped trajectory disappears. However, the axial trajectory is locally concave and presents a banana-like shape, as shown in Fig. 3c and d. The operating state of the rotor bearing system under 3000 rpm fluctuates slightly compared with that under 3500 rpm. In addition, the maximal displacement of the axial at *x* direction obviously increases when rotating speed increases between 2500 and 3500 rpm. When rotating speed of single-span rotor bearing system with spindle bending increases form 500 to 3500 rpm, the axial trajectory roughly exhibits three shapes. The displacement of the rotor bearing system increases in the *y*-direction firstly and then in the *x*-direction to reflect the change of maximum displacement from local to full circle. From the comparison of the main wave and harmonic wave, it is found that the unstable disturbance is most obvious around a rotating speed of 2500 rpm. By checking the vibration velocity of spindle in x-direction, it can be seen that the extreme vibration velocity is increasing with the increasing rotational speed.

**Figure 4:**
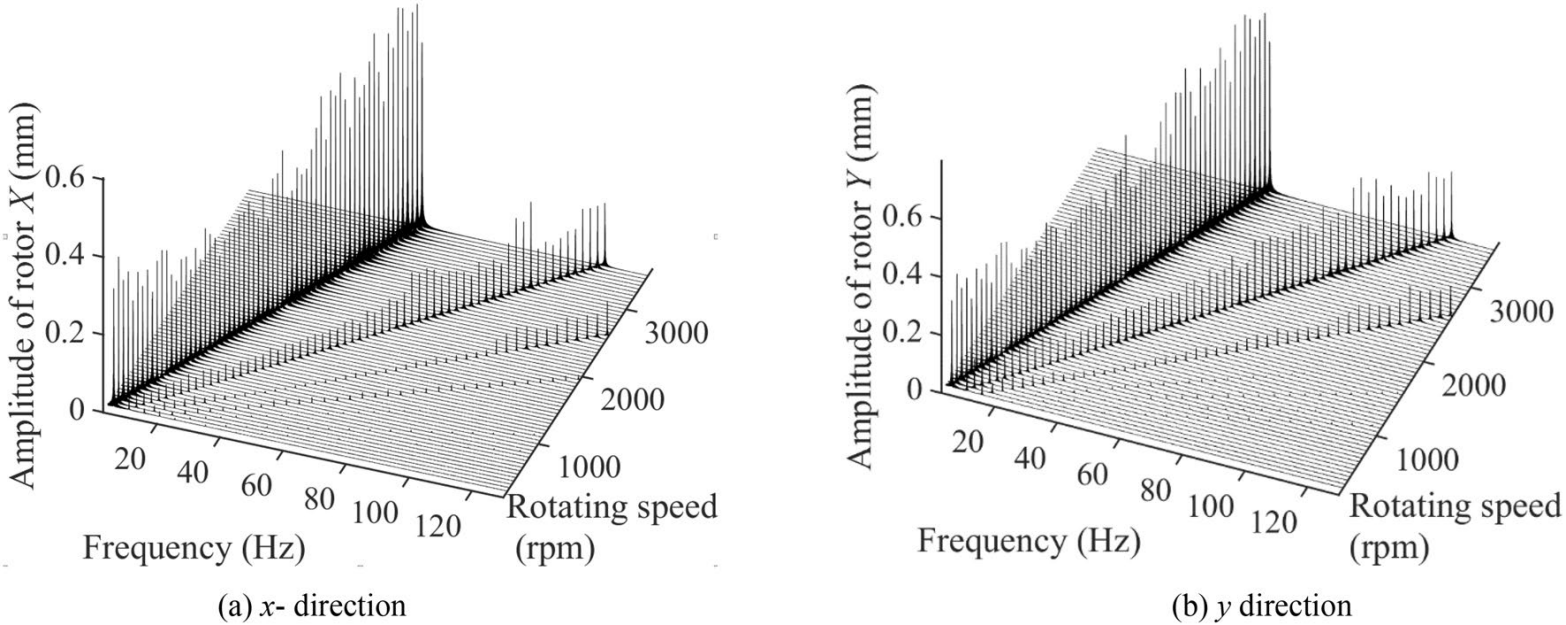
The three-dimensional spectrum diagram in (**a**) *x* (left) and (**b**) *y* (right) direction of single-span rotor bearing system with spindle bending under 300–3500 rpm.

Figure 4 depicts the three-dimensional spectrum diagram in *x* and *y* direction of single-span rotor bearing system with spindle bending under 300–3500 rpm. The system vibration is mainly influenced by 1×, 2× and 3× frequency component excitations. The frequency of 1× component excitation is approximately equal to the system’s rotation frequency. Meanwhile, the 1× component excitation plays a role of main vibration excitation sources. The amplitudes of 1× component excitation at *x* and *y* directions slightly decrease and then increase as the rotating speed increases from 300 to 3500 rpm. The amplitudes at *x* and *y* directions, respectively, varied among 0.2288–0.6029 mm and 0.2635–0.6656 mm. The change of amplitudes generated by 2× component excitation at *x* and *y* directions is similar to that generated by 1× component excitation as the rotating speed increases. However, the sudden increase in amplitudes occurs when rotating speed is around 3000 rpm. The amplitudes at *x* and *y* directions, respectively, varied among 0.02940–0.2250 mm and 0.05627–0.3112 mm. The amplitude generated by 3× frequency component excitation is much smaller than that generated by 1× and 2× frequency component excitation. The 3× frequency component excitation generates *x*-direction amplitude disturbance when rotating speed is large than 2050 rpm. However, it generates obvious *y*-direction amplitude disturbance under all rotating speeds.

### Mechanical response of spindle crack with no end loading

**Figure 5:**
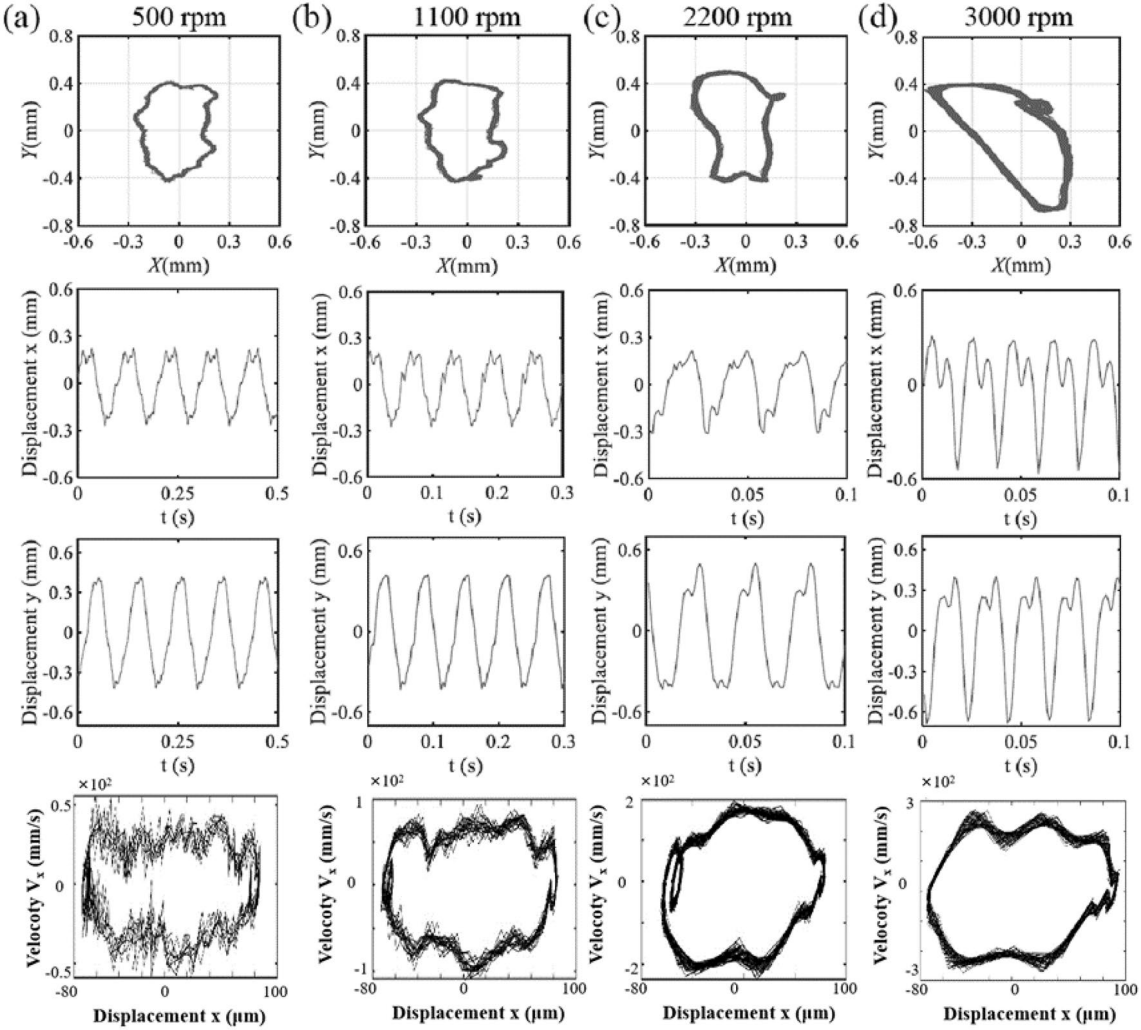
The axis trajectory, time history curve in *x*, *y* direction and vibration velocity in *x* direction of single-span rotor bearing system with spindle crack and no end loading under (**a**) 500 rpm, (**b**) 2500 rpm, (**c**) 3000 rpm and (**d**) 3500 rpm.

Figure 5 presents the axis trajectory and time history curve in *x*, *y* direction of single-span rotor bearing system for spindle bending with no end loading under 500 rpm, 1100 rpm, 2200 rpm and 3000 rpm. The rotor bearing system with spindle crack fault not only shows the obvious system instability, but also exhibits full-circle large sway degree under relatively low rotating speed. This is reflected by the continuously fluctuating axial trajectory in the radial direction and the obvious harmonic inclusions of time history curve at non peak and valley points as shown in Fig. 5a and b. This is because the crack appearance reduces the stiffness of the spindle itself. The opening and closing of crack cause the high-frequency vibration. When system’s rotating speed is around 1100 rpm, the local 8-shaped trajectory appears as shown in Fig. 5a and b, which is caused by spindle bending under high rotating speed operation. However, the amplitude of the axis does not increase significantly in any direction. The high-frequency harmonic disturbances are pronounced in the *x*-direction when system’s rotating speed is less than 1100 rpm.

If the system’s rotating speed increases to 2200 rpm, the high-frequency harmonic disturbances in *y*-direction enhance as shown in Fig. 5c. Meanwhile, the amplitude of the axis in *y*-direction increases. In addition, the local 8-shaped trajectory appears in other positions of axial trajectory. The axis trajectory is generally presented as a rectangle. When the system’s rotating speed continues to increase as shown in Fig. 5d, the high-frequency harmonic disturbances enhance in *x* and *y*-direction at the same time. The axial trajectory appears as a semicircle and retains the local 8-shaped trajectory in its original position. The axis exhibits linear drift in some stages. This may be in the closing stage of the spindle crack, thereby reducing the axial vibration stroke. When rotating speed of single-span rotor bearing system with spindle crack increases form 500 to 3000 rpm, the axial trajectory roughly exhibits four shapes. The displacement of the rotor bearing system obviously increases in *x* and *y*-direction when rotating speed is larger than 2200 rpm. From the comparison of the main wave and harmonic wave, it is found that the high frequency disturbance is most obvious at *x*-direction. When the system is operating under relatively high rotating speed as shown in Fig. 5c and d, the high frequency disturbance in the *y*-direction is also relatively enhanced. Compared to vibration velocity in x-direction of shaft with spindle bending, spindle crack with no end loading exhibits a more disturbed distribution of vibration velocity, but its vibration velocity still increases with the increase of rotational speed. And In the region of high rotational speed, an inner ring appears in the variation of vibration velocity with displacement.

**Figure 6:**
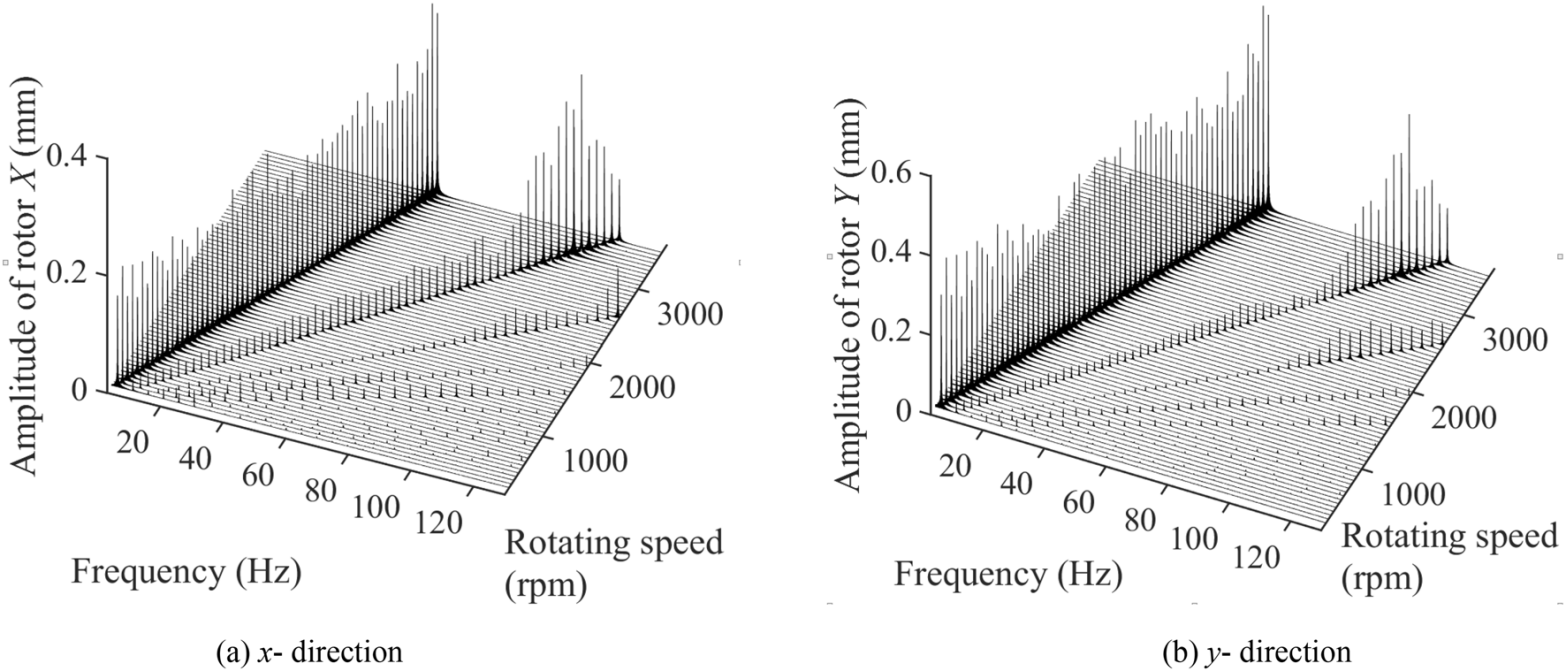
The three-dimensional spectrum diagram in (**a**) *x* (left) and (**b**) *y* (right) direction of single-span rotor bearing system with spindle crack and no end loading under 300–3500 rpm.

Figure 6 depicts the three-dimensional spectrum diagram in *x* and *y* direction of single-span rotor bearing system with spindle crack and no end loading under 300–3500 rpm. The system vibration is mainly influenced by 1×, 2×, 3× and even 4×, 5× frequency component excitation. The stiffness of the spindle is small when the crack opens, but large when it closes, resulting in nonlinear stiffness of the rotor and the occurrence of higher frequency component excitations. The frequency of 1 × component excitation is approximately equal to the system’s operating frequency. The change in rotating speed, except for the resonance rotating speed, has little impact on amplitude fluctuations. The amplitudes at x and y directions, respectively, varied among 0.2511–0.5245 mm and 0.1329–0.3325 mm. The resonance rotating speeds of 1× component excitation in *x* and *y* direction are around and larger than 3500 rpm. The amplitudes of 2× component excitation at *x* and *y* directions slightly increase from 300 to 2850 rpm. When the rotating speed is larger than 2850 rpm, 2× resonance occurs. And the amplitudes of 2× component excitation reach the largest value under 3250 rpm. The amplitudes at *x* and *y* directions, respectively, varied among 0.0194–0.3059 mm and 0.0114–0.4069 mm. The 3× component excitation generates obvious vibration amplitude when system reaches 3× resonance and the rotating speed is larger than 1850 rpm. The amplitudes of 3× component excitation reach the largest value around 2250 rpm. The amplitudes at *x* and *y* directions, respectively, varied among 0–0.0856 mm and 0–0.0892 mm. The 4× and 5× frequency component excitations generated a relatively large system’s vibration amplitude at *x* direction when rotating speed is less than 1250 rpm. However, the system’s vibration amplitude at *y* direction is relatively stable under any rotating speed.

### Mechanical response of spindle crack with end loading

**Figure 7:**
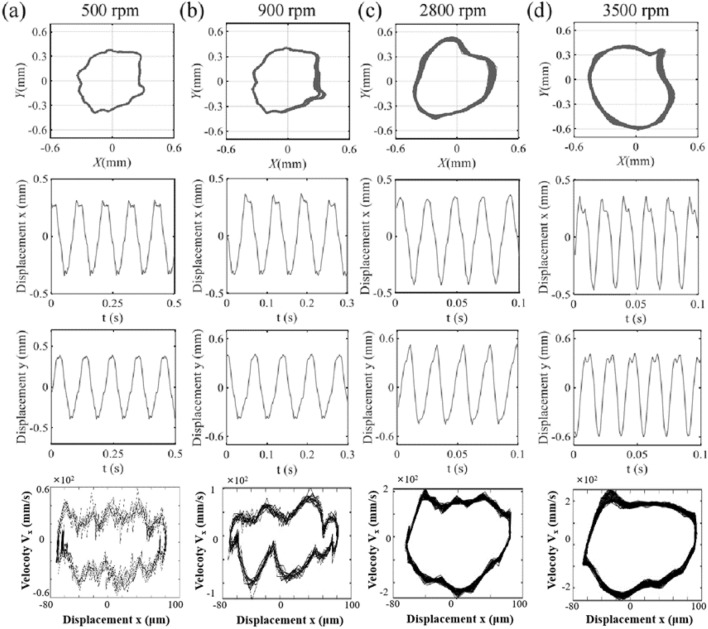
The axis trajectory and time history curve in *x*, *y* direction of single-span rotor bearing system with spindle crack and end loading under (**a**) 500 rpm, (**b**) 2500 rpm, (**c**) 3000 rpm and (**d**) 3500 rpm.

Figure 7 presents the axis trajectory and time history curve in *x*, *y* direction of single-span rotor bearing system with spindle crack and end loading under 500 rpm, 900 rpm, 2800 rpm and 3500 rpm. The rotor bearing system with spindle crack fault with end loading also exhibits full-circle sway degree under relatively low rotating speed as shown in Fig. 7a and b. The end load causes the spindle to be subjected to axial force, thereby increasing the overall stiffness, resulting in a decrease in full-circle sway degree. Moreover, the axis trajectory is closer to the circular or elliptical shape of normal operating conditions. In addition, the disturbance components decrease, and appearing high-frequency disturbances generally appear at the peaks and valleys of the waves. The amplitude difference in *x* and *y* directions is not significant, but the high-frequency disturbances in *x* direction are more pronounced. When the rotating speed increases to 900 rpm, the axis trajectory exhibits a sudden increase in amplitude over time. This may be caused by the sudden substantial opening or closing of cracks. As the rotating speed increases, the amplitude in *x*-direction of the single-span rotor bearing system with spindle crack and end loading increases.

When the rotating speed increases around 2800 rpm, the axis trajectory is locally concave and presents a banana-like shape, as shown in Fig. 7c. The amplitude in the *x* and *y* direction of the single-span rotor bearing system increases at the same time. When the rotating speed increases around 3500 rpm, the axis trajectory is locally convex as shown in Fig. 7d. However, the amplitude in the *x* and *y* direction did not increase significantly as rotating speed increases from 2800 to 3500 rpm. When rotating speed of single-span rotor bearing system of crack with end loading increases from 500 to 3500 rpm, the axial trajectory roughly exhibits three shapes. The amplitude of the rotor bearing system obviously increases in *x* and *y*-direction when rotating speed increases from 900 to 2800 rpm. From the comparison of the main wave and harmonic wave, it is found that the high frequency disturbance is most obvious at *x*-direction. When end loading is applied to the cracked spindle, the inner ring in vibration velocity plot illustrated in Fig. 5 disappears, but its vibration velocity increases with the increasing rotational speed.

**Figure 8:**
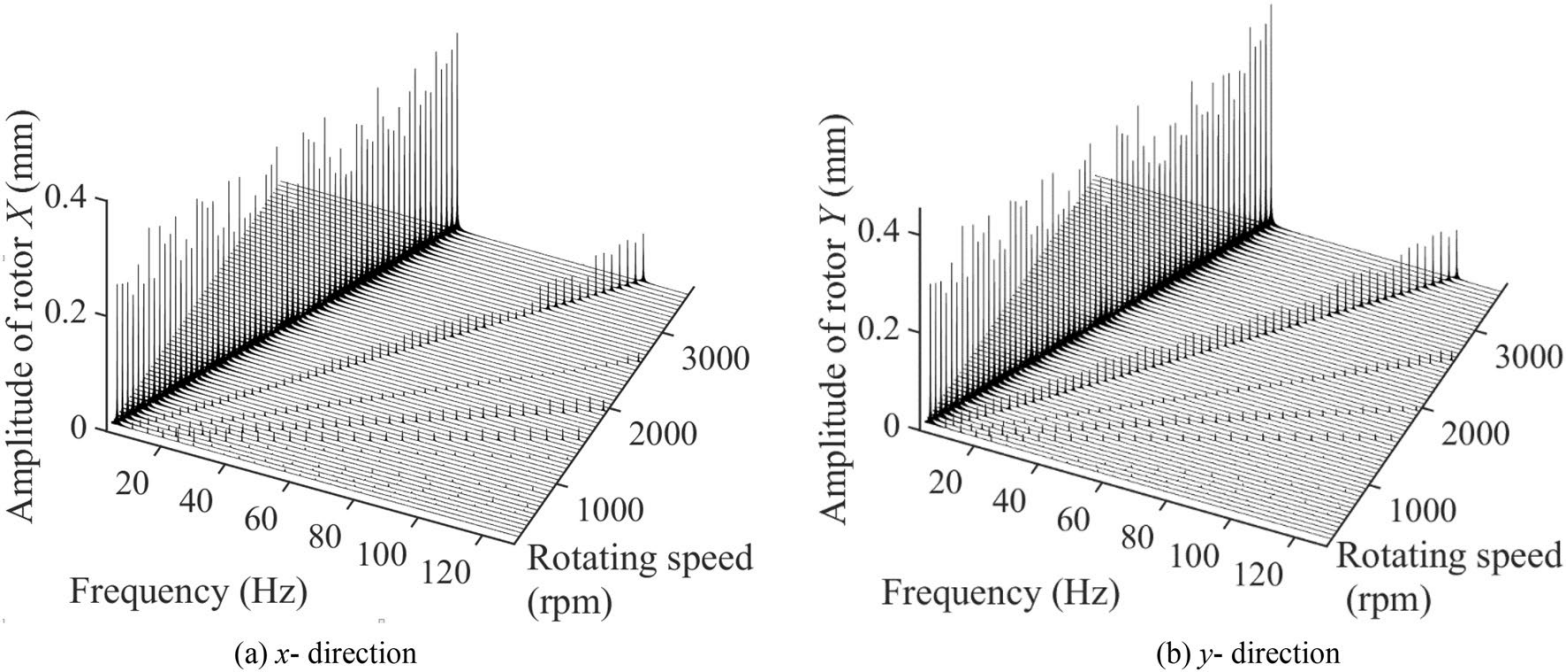
The three-dimensional spectrum diagram in (**a**) *x* (left) and (**b**) *y* (right) direction of single-span rotor bearing system with spindle crack and end loading under 300–3500 rpm.

Figure 8 presents the three-dimensional spectrum diagram in x and y direction of single-span rotor bearing system with spindle crack and end loading under 300–3500 rpm. The system vibration is mainly influenced by 1×, 2×, 3×, 4× and 5× frequency component excitations. The frequency of 1× component excitation is approximately equal to the system’s operating frequency. The amplitudes at *x* and *y* directions generated by 1× frequency component excitations fluctuate steadily as the system’s rotating speed increases. The amplitudes at *x* and *y* directions, respectively, varied among 0.2087–0.3432 mm and 0.2501–0.4548 mm. The amplitudes in *x* and *y* directions generated by 2× component excitation increases from 300 to 3500 rpm in fluctuations. The amplitudes at *x* and *y* directions, respectively, varied among 0.0099–0.0845 mm and 0.0228–0.1068 mm. Different from other faults, the amplitudes at *x* and *y* directions generated by 3× frequency component excitation are smaller than that generated by 4× and 5× frequency component excitations. At *x* direction, the amplitudes of 3× frequency component excitation decreases as the system’s rotating speed increases. However, it increases at y direction. The amplitudes at *x* and *y* directions, respectively, varied among 0.0039–0.0160 mm and 0.0101–0.0246 mm. The amplitudes at *x* and *y* directions generated by 4× and 5× frequency component excitations fluctuate steadily as the system’s rotating speed increases. Moreover, the amplitude generated by 4× frequency component excitation is roughly the same as that generated by 5× frequency component excitation. For 4× frequency component excitation, the amplitudes at *x* and *y* directions, respectively, varied among 0.0159–0.0258 mm and 0.0090–0.0214 mm. For 5× frequency component excitation, the amplitudes at *x* and *y* directions, respectively, varied among 0.0138–0.0208 mm and 0.0120–0.0171 mm.

## Fault diagnosis by CNN

By the vibration response analysis based on experiment, the shaft trajectory under spindle bending shows three shapes with the increase of rotational speed, the local 8-shaped trajectory appears under 2500 rpm, and only at this time there is a harmonic interference in *x* and *y* directions, and the maximum displacement in both directions continues to increase under 3000 rpm. while the appearance of crack reduces the shaft stiffness, and the crack opening and closing during rotation causes high frequency vibration, which makes the harmonic disturbance in *x* direction more obvious, and the local 8-shaped trajectory occurs at both medium and high rotational speeds, and the maximum displacement in both directions is basically the same below 3000 rpm. However, when end load is applied to the cracked rotor, the spindle is subjected to axial force, which increases the overall composite stiffness, and at this time, the high-frequency disturbance decreases again, and the harmonics occur in both *x* and *y* directions only when the rotational speed is higher than 2200 rpm. Consistent with the cracked spindle without end loading, the maximum displacements in both directions are basically identical when the rotational speed is lower than 3000 rpm.

It is obvious that the rotor-bearing systems corresponding to the three faults may have similar trends in vibration, and a more reliable basis for discrimination is the combination of maximum displacement in *x*, *y* direction, harmonic disturbances, and shaft trajectory. Compared to random forests^33^, support vector machines^34^, deep belief nets (DBNs)^35^ and so on, Convolutional Neural Network (CNN), as a machine learning method, is characterized by learning through layer-by-layer transmission. It is also capable of recognizing important features such as excitation of each multi-frequency component caused by spindle crack faults. In other words, it can extract higher level features such as harmonic disturbances and capture higher level semantic information that can be utilized to accurately diagnose failure.

Convolutional neural network (CNN) belongs to feed-forward neural network, which belongs to supervised mode of deep learning model and consists of input layer, convolutional layer, pooling layer, fully connected layer and output layer. The convolutional layer and pooling layer, as the core of 1D CNN, extracts the input features by convolution kernel to capture their relevance, and performs the extraction of local features from input data by convolution layer through convolution kernel according to the specified step size. The convolution process can be represented as

1 $$Y_{j}^{k} = X\left( {\sum Z_{ij}^{k} W_{i}^{k - 1} + A_{j}^{k} } \right)$$

where ${Y}_{j}^{k}$ and ${A}_{j}^{k}$ are the output and bias of the neuron, respectively; ${Z}_{ij}^{k}$ is the *ij*th convolutional kernel of layer *k*; *k* shows the layer *k* of network; and X(·) represents the activation function.

In 1D CNN, the local connectivity strategy is inspired by biological visual system, in which a single neuron is connected to a small localized region of input. Which not only reduces the parameter count of model, also improves the computational efficiency and the generalization ability. The weight sharing strategy further reduces the number of parameters and it allows the network to detect the same features at different locations.

The pooling layer is another key component in CNNs that is responsible for dimensionality reduction and feature compression. By taking the maximum (maximum pooling) or average (average pooling) value within a spatial neighborhood, the pooling operation is able to reduce the spatial dimensionality, which decreases the computational complexity and increases the model robustness. The maximum pooling mode can be calculated as

2 $$y_{i,j,k} = max\left( {x_{i + p,j + q,k} } \right)$$

where ${y}_{i,j,k}$ is the output feature map; ${x}_{i+p,j+q,k}$ shows the feature map of *k*th channel from the $i+p$ row, $j+p$ column; and *p* and *q* indicate the coordinate steps within pooling window. After convolution and pooling, the spatial structure of data has been fully extracted and compressed, the fully connected layer is responsible for integrating these features and outputting predictions. Mapping to normalized intervals is used for classification through logical activation functions. The logical representation of full connected layer is given by

3 $$y_{{\mathbf{o}}} = f\left( {\mathop \sum \limits_{i = 1}^{n} \omega_{i} x_{i} + b} \right)$$

where *x*_*i*_ is the *k*th feature vector of input; *y*_*0*_ shows the feature mapping of output; *n* gives the dimension of input features; *ω*_*i*_ represents the weight corresponding to each input vector and which is a learnable parameter; and *f*(∙) is the logical activation function.

Due to the differences in mechanical response mentioned above, three types of faults are classified and identified by convolutional neural network. The testing data of each fault contains the signals generated by single-span rotor bearing system under the rotating speed of 300–3500 rpm to eliminate the speed impact on fault identification. The collection time at each rotating speed lasts for 30 s to ensure the authenticity and validity of the data in the time dimension. Before network training, the data are pre-processed to meet the input format of convolutional neural network. Each group of data is segmented with a sample time of 1 s, and the samples are labelled with the corresponding fault conditions. For different fault conditions, 20 1-s-long signal sequences are taken as input signal samples after normalization for each speed. The number of signal samples are 4140. For 1D CNN, which consists of two convolutional layers, two pooling layers, and two fully-connected layers with batchsize of 100. ADAM optimization method is adopted with an initial learning rate of 0.001, and the final classification of the samples is outputted from classification layer with a statistical accuracy. And the structure and hyperparameters of 1D CNN for each layer are shown in Table 2. In addition, we use 80% of the samples as the training set, 10% of the samples as the validation set, and the remaining 10% of the samples as the test set to verify the diagnostic effectiveness of the model for fault diagnosis of single-span rotor bearing system.

**Table 2:** The structure and hyperparameters of 1D CNN for each layer.

Number	Convolutional layers		Activation function	Pooling layers		Fully connected layers	Learning rate
	Kernel size	Number of kernels	Type	Size	Stride	Size	–
1	32	64	Relu	8	2	–	0.001
2	16	32	Relu	4	2	–	
3	–	–	–	–	–	10	
4	–	–	–	–	–	3	
5	Softmax layer						
6	Classification layer						

**Figure 9:**
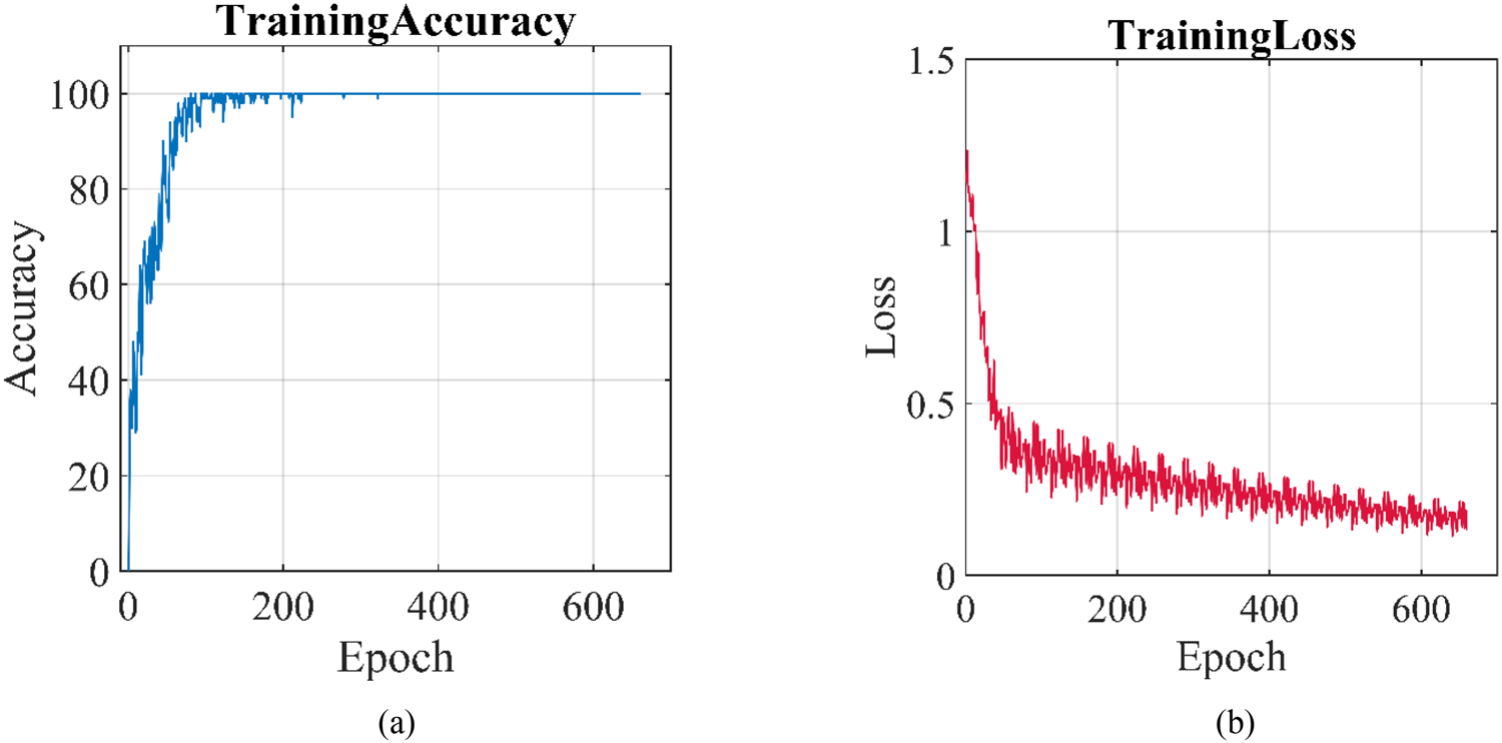
(**a**) The training accuracy and (**b**) training loss curves during CNN training period.

**Figure 10:**
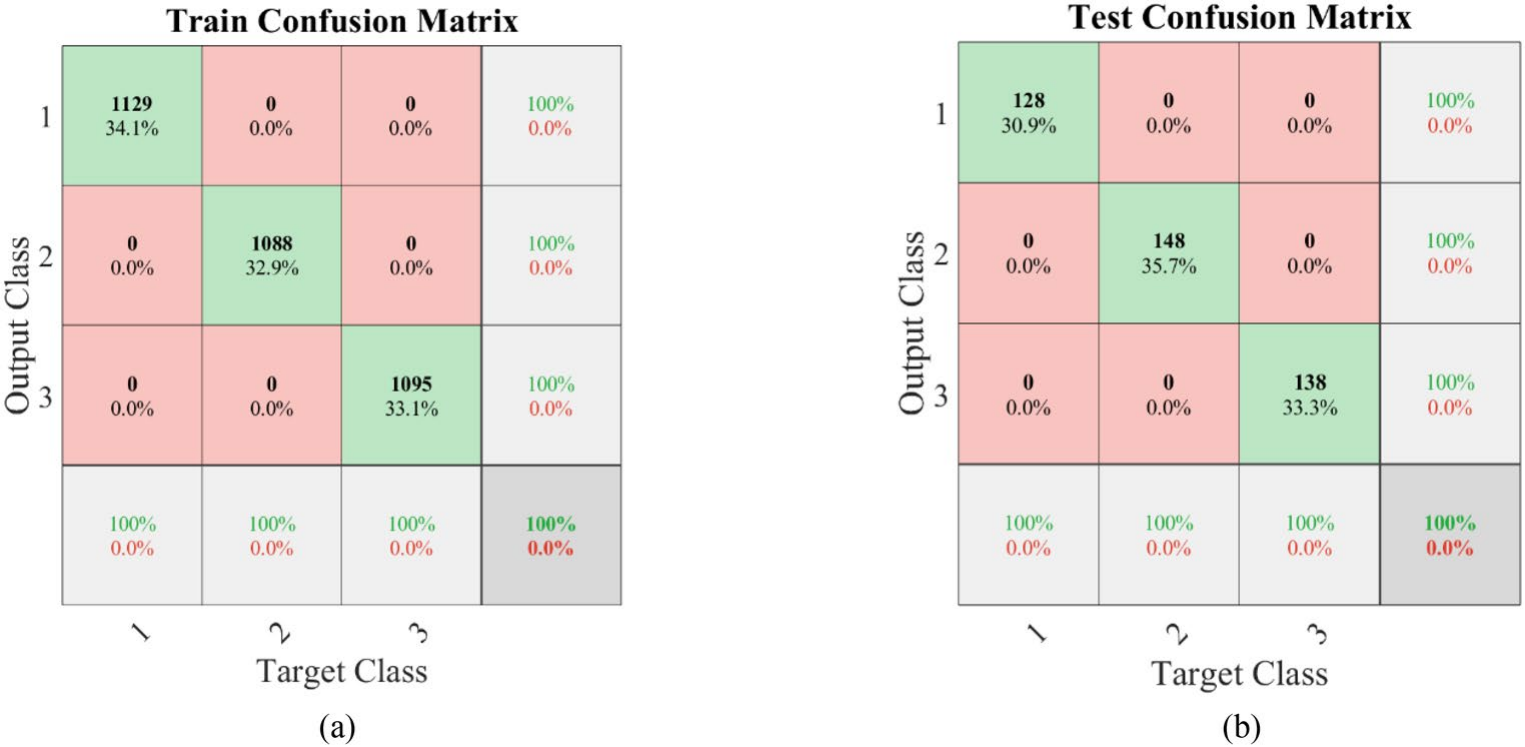
(**a**) The train confusion matrix and (**b**) testing confusion matrix of machine learning of our CNN network. 1, 2 and 3 respectively represent the spindle crack fault with end loading, spindle crack fault without end loading and spline bending fault.

The 1D-CNN expansion is used to classify signals. The CNN model consists of two convolutional pooling blocks composed of one-dimensional convolutional layers and pooling layers, two fully connected layers, and a Softmax output layer. Relu serves as the activation function and Adam serves as the optimization algorithm. Each training batch is set to 100, and the total number of training epochs is set to 20. The training accuracy curve and loss curve during the training process are shown in Fig. 9a and b, respectively. As the training frequency increases, the accuracy increases rapidly, while, the loss also rapidly decreases. When the training frequency reaches 100 times, the accuracy is close to 100%. As the training frequency continues to increase, the accuracy gradually stabilizes and maintains at 100%. The resulting classification confusion matrix is shown in Fig. 10. In Figs. 1, 2 and 3 respectively represent the spindle crack fault with end loading, spindle crack fault without end loading and spline bending fault. The sample distribution of three types of faults (spindle bending, spindle crack without end loading and spindle crack with end loading) in training set is essentially uniform, accounting for 34.1%, 32.9%, and 33.1% of the training set, respectively. And the confusion matrices for CNN training and testing demonstrate the percentage of three types of faults as 34.1%, 32.9%, 33.1% and 30.9%, 35.7%, 33.3%, respectively. It seems that the samples of three faults do not differ drastically during diagnosis using CNNs, and therefore, the effect of a slight imbalance in dataset on fault classification is ignored. From Fig. 10, it can be seen that the sample distribution of the three types of faults in the training set is uniform, accounting for 34.1%, 32.9%, and 33.1% of the training set, respectively. The classification accuracy of the training set and the test set for the three types of working conditions is 100%, indicating that CNN has a good classification effect for the spindle crack fault with end loading, spindle crack fault without end loading and spline bending fault. This indicates that our network has good stability and can significantly identify signal features under different operating conditions so as to make effective fault classification. Of course, many defects often occur simultaneously in actual detection requirements. The above fault diagnosis reflects that machine learning based on neural networks has good identification capabilities based on different signal characteristics. Thus, multiple-fault data must be obtained through experimental testing to expand above training set to strengthen the identification capability and make it practical.

## Conclusion

In this paper, the vibration mechanical response of the single-span rotor bearing system is investigated by experiment. The characteristics about the system with three faults of spindle bending, spindle crack without end loading and spindle crack with end loading are analysed. In addition, the fault diagnosis is conducted by CNN machine learning. The conclusions can be drawn as follows:

1. For spindle bending fault, the axial trajectory roughly exhibits three shapes form 500 to 3500 rpm. The displacement of the rotor bearing system increases in the *y*-direction firstly and then in the *x*-direction to reflect the change of maximum displacement from local to full circle. From the comparison of the main wave and harmonic wave, it is found that the unstable disturbance is most obvious around a rotating speed of 2500 rpm. The system vibration is mainly influenced by 1×, 2× and 3× frequency component excitations.
2. For spindle crack fault without end loading, the axial trajectory roughly exhibits four shapes. The displacement of the rotor bearing system obviously increases in the *x* and *y* direction when rotating speed is larger than 2200 rpm. From the comparison of the main wave and harmonic wave, it is found that the high frequency disturbance is most obvious at *x*-direction. When the system is operating under relatively high rotating speed as shown in Fig. 5c and d, the high frequency disturbance in *y*-direction is also relatively enhanced. The system vibration is mainly influenced by 1×, 2×, 3× and even 4×, 5× frequency component excitations. When increase end loading, the axial trajectory roughly exhibits three shapes. The amplitude of the rotor bearing system obviously increases in the x and y-direction when rotating speed increases from 900 to 2800 rpm. From the comparison of the main wave and harmonic wave, it is found that the high frequency disturbance is most obvious at *x*-direction.
3. When the training frequency reaches 100 times, the accuracy is close to 100%. As the training frequency continues to increase, the accuracy gradually stabilizes and maintains at 100%. the sample distribution of the three types of faults in the training set is uniform, accounting for 34.1%, 32.9%, and 33.1% of the training set, respectively. The classification accuracy of the training set and the test set for the three types of working conditions is 100%, indicating that CNN has a good classification effect for the spindle crack fault with end loading, spindle crack fault without end loading and spline bending fault.

## Data Availability

Data is provided within the manuscript or supplementary information files.
